# From Natural Hosts to Agricultural Threats: The Evolutionary Journey of Phytopathogenic Fungi

**DOI:** 10.3390/jof11010025

**Published:** 2025-01-01

**Authors:** Asanka Madhushan, Dulan Bhanuka Weerasingha, Evgeny Ilyukhin, Paul W. J. Taylor, Amila Sandaruwan Ratnayake, Jian-Kui Liu, Sajeewa S. N. Maharachchikumbura

**Affiliations:** 1 School of Life Science and Technology, Center for Informational Biology, University of Electronic Science and Technology of China, Chengdu 611731, China; asankakwm@gmail.com (A.M.); weerasinghawadb@gmail.com (D.B.W.); 2 Laboratory of Plant Pathology, Swift Current Research and Development Centre, Agriculture and Agri-Food Canada, Swift Current, SK S9H 3X2, Canada; evgeny.ilyukhin@gmail.com; 3 Faculty of Science, The University of Melbourne, Parkville, VIC 3010, Australia; paulwjt@unimelb.edu.au; 4 Department of Applied Earth Sciences, Faculty of Applied Sciences, Uva Wellassa University, Passara Road, Badulla 90000, Sri Lanka; as_ratnayake@uwu.ac.lk

**Keywords:** emerging strains, resistance, Sordariomycetes, virulence

## Abstract

Since the domestication of plants, pathogenic fungi have consistently threatened crop production, evolving genetically to develop increased virulence under various selection pressures. Understanding their evolutionary trends is crucial for predicting and designing control measures against future disease outbreaks. This paper reviews the evolution of fungal pathogens from natural habitats to agricultural settings, focusing on eight significant phytopathogens: *Pyricularia oryzae*, *Botrytis cinerea*, *Puccinia* spp., *Fusarium graminearum*, *F. oxysporum*, *Blumeria graminis*, *Zymoseptoria tritici*, and *Colletotrichum* spp. Also, we explore the mechanism used to understand evolutionary trends in these fungi. The studied pathogens have evolved in agroecosystems through either (1) introduction from elsewhere; or (2) local origins involving co-evolution with host plants, host shifts, or genetic variations within existing strains. Genetic variation, generated via sexual recombination and various asexual mechanisms, often drives pathogen evolution. While sexual recombination is rare and mainly occurs at the center of origin of the pathogen, asexual mechanisms such as mutations, parasexual recombination, horizontal gene or chromosome transfer, and chromosomal structural variations are predominant. Farming practices like mono-cropping resistant cultivars and prolonged use of fungicides with the same mode of action can drive the emergence of new pathotypes. Furthermore, host range does not necessarily impact pathogen adaptation and evolution. Although halting pathogen evolution is impractical, its pace can be slowed by managing selective pressures, optimizing farming practices, and enforcing quarantine regulations. The study of pathogen evolution has been transformed by advancements in molecular biology, genomics, and bioinformatics, utilizing methods like next-generation sequencing, comparative genomics, transcriptomics and population genomics. However, continuous research remains essential to monitor how pathogens evolve over time and to develop proactive strategies that mitigate their impact on agriculture.

## 1. Introduction

**Figure 1:**
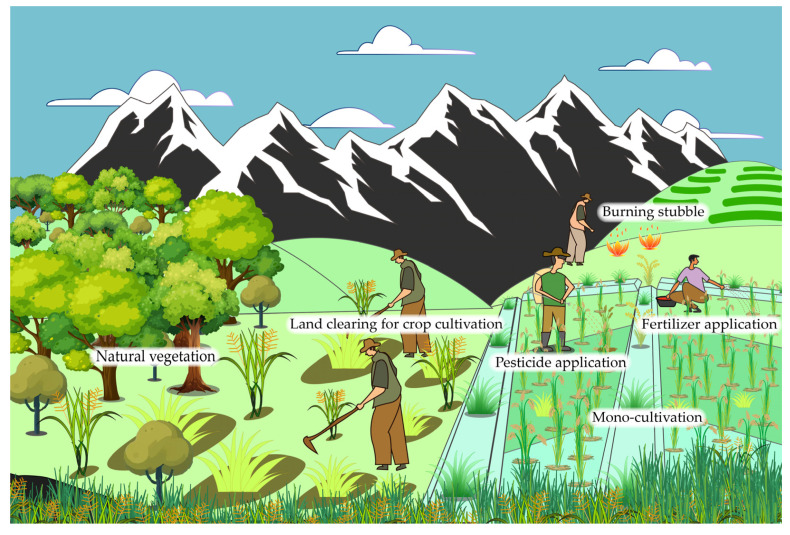
An image illustrating human-induced selection pressures in an agricultural ecosystem. Farming practices such as monocropping, pesticide application, fertilization, and stubble burning affect the evolutionary dynamics of pathogenic fungi. Expanding crop cultivation into natural ecosystems allows pathogenic fungi from natural settings to transfer to cultivated crops, where they may undergo rapid evolutionary changes due to exposure to previously unexperienced conditions in agricultural lands.

By transitioning from nomadic to settled lifestyles, growing food plants, and rearing animals, humans established a stable food supply, allowing for year-round sustenance. These plants evolved into crops through long-term domestication, which involved selecting desired traits, both sexual and asexual reproduction over multiple generations, and gradually shaping the genetic variation within local populations [1,2]. Therefore, domesticated crop plants are genotypically and phenotypically different from their wild ancestors. They are cultivated on a large scale under intensive management within agroecosystems. Compared to natural ecosystems, which are the habitats of wild plants, agroecosystems usually have a greater possibility of being affected by pathogens with higher aggressiveness [3]. In agricultural crop fields, the combination of high plant density, limited genetic diversity (i.e., monoculture), high nutrient availability [3,4], and the absence of natural enemies creates a stable and predictable environment for pathogens. Moreover, in contrast to natural ecosystems, pathogens in agricultural ecosystems are exposed to a variety of human-induced selection pressures, including the selection for specific virulence strategies driven by host defenses, recurring cycles of extinction and re-colonization influenced by host dynamics (e.g., resistant cultivars), chemical applications (e.g., agro-chemicals), and changing cultural practices (e.g., crop rotations and stubble burning), all of which contribute to shaping their evolutionary trajectories [5] (Figure 1).

In agroecosystems, three evolutionary trends posing new severe issues have been identified: (1) the evolution or adaptation of pathogens causing previously unreported diseases, (2) the emergence of strains capable of overcoming host resistance mechanisms, and (3) the development of pathogen resistance against chemicals used in disease control [6]. Generally, pathogenic organisms are evolving rapidly compared to their host plants, and they also play an important role in the natural selection of host plants [7]. There are several mechanisms proposed for the emergence of pathogens in agroecosystems, including (1) co-evolution of the pathogen along with the host plant (host-tracking), (2) pathogen infection of a new host where the disease is not previously reported (host shift or host jump), and (3) development of virulence within the pathogen by transferring genes or genomic regions from other species [4]. However, the evolution of plant diseases is a continuous process, and it cannot be halted. What humans can do is understand how and why diseases occur and implement possible remedies early to overcome economic and ecological losses.

Studying the evolution of plant diseases and understanding how pathogens domesticate from natural habitats to agricultural ecosystems are valuable for developing holistic approaches to disease management that consider both the ecological factors influencing disease spread and the evolutionary processes shaping pathogen populations [8]. This will improve disease management strategies by predicting outcomes and identifying sources of pathogen virulence, aiding in sustainable agricultural practices. Such knowledge is essential for optimizing disease-resistance gene management in crops and supporting resistance breeding programs [9,10]. Additionally, it helps identify biotic threats to ecosystems and plan conservation strategies. Understanding plant disease history also informs phytosanitary regulations to prevent pathogen spread via global trade [11].

Among the disease-causing microorganisms, fungi comprise some of the most devastating pathogens [12,13]. Many phytopathogenic fungi have been found to cause massive damage to agricultural produce, including yield loss, food and feed contamination, and crop failure [12,14]. Therefore, this review focuses on fungal plant pathogens and how they domesticate from natural habitats to agricultural ecosystems. We have selected eight fungal pathogens: *Pyricularia oryzae*, *Botrytis cinerea*, *Puccinia* spp. *Fusarium graminearum*, *F. oxysporum*, *Blumeria graminis*, *Zymoseptoria tritici* and *Colletotrichum* spp. These pathogens are among the most devastating to agricultural production, as described by Dean et al. [15], in their listing of the top ten fungal pathogens in molecular plant pathology. We excluded *Ustilago maydis* and *Melampsora lini*, ranked 9th and 10th on the list, as they are of greater scientific significance than economic importance. All the selected fungi cause significant economic losses, particularly on major food crops worldwide. The selected pathogens represent major phytopathogenic fungal groups, primarily Sordariomycetes (*Colletotrichum* spp., *F. graminearum*, *F. oxysporum*, and *Py. oryzae*), followed by Leotiomycetes (*Bo. cinerea* and *Bl. graminis*), Dothideomycetes (*Z. tritici*), and Pucciniomycetes (*Puccinia* spp.). Additionally, they encompass different pathogenic fungal lifestyles, including necrotrophy (*Bo. cinerea, Colletotrichum* spp., *Fusarium* spp., *Py. oryzae,* and *Z. tritici*), hemibiotrophy (*Colletotrichum* spp.), and biotrophy (*Bl. graminis* and *Puccinia* spp.). This review explores the evolutionary forces and genetic changes that drive their adaptation to dynamic agroecosystems, while also detailing their wild and domesticated host range. Drawing insights from these pathogens, we further discuss possible ways to retard pathogen evolution, along with modern techniques used in understanding evolutionary mechanisms.

## 2. Evolutionary Journey of *Pyricularia oryzae*

The fungus *Pyricularia oryzae* (Syn. *Magnaporthe oryzae*), previously known as *Magnaporthe grisea* [16] causes blast disease in several wild and domesticated poaceous hosts. This necrotrophic ascomycete belongs to the family Magnaporthaceae (Magnaporthales) [17,18]. According to the associated host plant, there are different pathotypes of *Py. oryzae*, such as *Py. oryzae Oryza*, *Py. oryzae Triticum*, *Py. oryzae Eleusine*, *Py. oryzae Setaria*, and *Py. oryzae Lolium*, which are causing blast disease in rice (*Oryza sativa*), wheat (*Triticum aestivum*), finger millet (*Eleusine coracana*), foxtail millet (*Setaria italica*), and perennial ryegrass (*Lolium perenne*), respectively [19]. In addition, wild hosts of *Py. oryzae* include common weeds in rice agroecosystems, such as torpedo grass (*Panicum repens*) and cutgrass (*Leersia hexandra*) [20]. The occurrence of *Py. oryzae* in rice was first reported in China in 1637 as ‘rice fever disease’ [17,21], and in 1906, it was reported as ‘Blast’ in the USA [21]. However, the origin of blast disease in cultivated crops is doubtful, whether it occurred first in rice or other wild species [17].

Several studies have demonstrated that the emergence of *Py. oryzae* in agroecosystems occurred through host tracking (co-evolution) and shifting [21,22]. Host–pathogen co-evolution reflects the correspondence between the host center of origin and the pathogen center of origin [4]. Rice is believed to have originated around 7000 years ago in the Yangtze Valley in China from the wild relative *O. rufipogon* [23,24]. Setaria millet also originated in China and was domesticated and co-cultivated with rice [21,25]. *Pyricularia oryzae* is believed to have shifted from Setaria millet to rice as a result of the domestication process. The domestication of rice provided the opportunity for the blast pathogen to shift into the new host since the large homogeneous populations are one of the best targets for pathogenic migrants [21]. In species exhibiting both sexual and asexual reproduction, the sexual form is believed to be ancestral [26]. Building on this premise, Saleh et al. [22] also supported the Asian center of origin for both the pathogen and its host, identifying South China–Laos–North Thailand region as the likely center of origin for *Py. oryzae* due to the persistence of sexual reproduction and the presence of diverse populations. In many other areas of the world, *Py. oryzae* predominantly reproduces asexually, giving rise to pandemic clonal populations [27].

After specializing in rice, *Py. oryzae* has also undergone additional host shifts to weeds associated with rice, such as cutgrass (*Leersia hexandra*) and torpedo grass (*Panicum repens*) [21]. However, *Py. oryzae* lineages that are infecting non-rice hosts are found to be non- or poorly pathogenic on rice plants [17,21,28], possibly due to the abundance of avirulence gene AVR-Co39, which is less in rice infecting lineages [21]. *Pyricularia oryzae* was transferred into other economically important hosts, such as wheat, maize, barley, perennial ryegrass, etc., during the later distribution of rice cultivation worldwide [17,21]. For instance, in 1985, *Py. oryzae* was reported from wheat for the first time in Brazil and was expanded to South American wheat-growing areas [29,30]. However, the *Py. oryzae* strain responsible for wheat blast differs from the one causing rice blast [31,32]. Consequently, the wheat blast outbreak in North America in 2011 was attributed to an isolate that had shifted from ryegrass (*Lolium* spp.) [30,33].

As described above, *Py. oryzae* possesses prominent asexual reproduction with limited sexual reproduction that is confined to specific centers of origin. Therefore, the contribution of sexual recombination appears to be less significant, resulting in novel pathotypes. However, several mechanisms have been identified as contributing to genetic variations in asexual populations of *Py. oryzae*, facilitating adaptation to dynamic agricultural systems. Comparative genome analyses of *Py. oryzae* isolates have shown that chromosomal rearrangements play a pivotal role in host-specific adaptation, involving the gain and loss of certain genes [34,35]. For instance, the avirulence gene avr-Pita is tightly linked to a telomere on chromosome 3 of *Py. oryzae*, and the loss of chromosome tips has led to a shift in the pathogen from avirulence to virulence [36,37]. Mini-chromosomes, also referred to as supernumerary or accessory chromosomes, are also found to contribute to host adaptation of *Py. oryzae* [38,39], as they are enriched in transposons and repetitive elements and the presence of virulence-related genes [38,39,40,41]. These dispensable mini-chromosomes are present only in some, but not all individuals in a population, showing non-mendelian inheritance [42,43,44] and are hypothesized to have independently emerged from structural rearrangements of core-chromosomes [39]. Furthermore, studies have revealed that rapid variations in the effector repertoire within the fungal population, either through the loss of avirulence genes [17,45] or mutations (including point mutations, insertions, deletions and frame-shifts in avirulence genes) [17,36,46] result in the rapid adaptation of *Py. oryzae* to multiple resistant rice cultivars. Parasexual recombination, which involves the exchange of genetic material without the formation of sexual spores, occurs through the fusion of hyphae from different strains of the same species [47], and has also been found to create genetic variations in *Py. oryzae*, leading to enhanced virulence [47,48,49,50].

Moreover, in response to human-induced selection pressures in agroecosystems, *Py. oryzae* has been found to overcome host resistance [51] and acquire fungicide resistance [52,53,54,55,56,57,58,59,60], impeding the control efforts against blast disease.

## 3. Evolutionary Journey of *Botrytis cinerea*

*Botrytis cinerea* causes gray mold disease on economically important fruits, vegetables and ornamental plants worldwide [61,62]. This necrotrophic ascomycete fungus belongs to the family Sclerotiniaceae (Helotiales) [61,63]. There is no evidence for a precisely defined center of origin for *Bo. cinerea*, probably due to its adaptability to thrive wherever host plants are cultivated, ranging from tropical and subtropical regions to cold temperate zones, even in deserts [64]. The host range of *Bo. cinerea* is estimated to encompass 586 genera of vascular plants, though it is likely even broader, as there are limited reports of diseases in wild plants [64]. Several studies on the differentiation of *Bo. cinerea* populations based on host preferences have produced inconclusive findings, possibly due to variations in the study methods (see Leroch et al. [65]; Atwell et al. [66]; Kozhar et al. [67]). Fournier et al. [68] demonstrated that *Bo. cinerea* is a species complex composed of genetically distinct groups that may represent different cryptic species. However, Plesken et al. [69] reported the first unequivocal example of host specialization in *Bo. cinerea*, identifying *Bo. cinerea Iris* is highly aggressive on the monocot plant *Iris pseudacorus* leaves compared to other *Bo. cinerea* strains from different hosts. This could potentially reflect the co-evolution of *Bo. cinerea* with its host.

Studies have provided valuable insights into unveiling the broad host range of *Bo. cinerea* through the generation of novel genetic variations, resulting in increased virulence. Zhu et al. [70] discovered that *Bo. cinerea* acquired potential virulence genes from both plants and bacteria, highlighting the significant role of inter-kingdom horizontal gene transfer (HGT) in extending the host range of this plant pathogen. *Botrytis cinerea* has also been observed to acquire the bikaverin gene cluster (which is involved in the production of the red pigment bikaverin, known for its antibiotic and antitumor properties) from *Fusarium oxysporum* through HGT [71]. This acquisition may provide a protective mechanism for *Bo. cinerea*, potentially enhancing its ability to compete in its ecological niche and defend against predators and pathogens [72]. Additionally, accessory chromosomes have also been found to play a role in the host adaptation of *Bo. cinerea* [73]. Furthermore, the diversification and balancing selection in certain genes, such as endopolygalacturonase-encoding genes Bcpg1 and Bcpg2, may allow *Bo. cinerea* to evade recognition by the host immune system and delay the plant defense reactions during the early stages of infection [74].

*Botrytis cinerea* populations within agroecosystems exhibited strong localization, with minimal migrations even among neighboring fields [67]. Therefore, the genetic structure and diversity of these fungi have mainly been influenced by geographic factors and human-induced selection pressures [67]. Several studies have reported resistance of *Bo. cinerea* against commonly used fungicides for controlling gray mold disease in fields [61,75,76,77,78,79]. The rapid acquisition of fungicide resistance is due to its widespread distribution, large population sizes and mixed reproductive strategies [67]. Additionally, fungicide resistance in gray mold populations is described to occur through various genetic and biochemical mechanisms, including target site mutations, efflux pump overexpression, and metabolic detoxification [62,65,80,81]. However, Kozhar et al. [67] observed that there is no correlation between the resistance allele and genetic background, indicating de-novo development of fungicide resistance. Their findings suggest that the evolution of fungicide resistance is more likely attributed to frequent extinction and recolonization events involving diverse genotypes rather than the dispersion of resistance alleles across fields through the migration of a dominant genotype.

*Botrytis cinerea* exhibits both sexual and asexual reproduction. During sexual reproduction, *Bo. cinerea* develops a fruiting body called an apothecium containing ascospores, following the fertilization of asexual resting structures known as sclerotia that typically reside in plant residues in the topsoil layer for many years [66,82]. Sexual reproduction is noted to be a key contributor to the genetic variation in *Bo. cinerea* populations, primarily through sexual recombination [83,84,85]. However, the sexual state is not commonly observed in natural settings [86,87,88,89]. Consequently, in such instances, genetic variability occurs through heterokaryosis [87] or is suggested to be clarified through sexual or parasexual recombination [66]. In *Bo. cinerea*, asexual reproduction plays a vital role in the dispersal of clones (dry conidia) across field environments, with wind and turbulent air currents enabling long-distance spread and rain splash aiding in shorter-range distribution [87].

## 4. Evolutionary Journey of *Puccinia* spp.

*Puccinia* spp. are obligate biotrophs belonging to the family Pucciniaceae (Uredinales) [90,91]. Species of *Puccinia* have been found to cause rust diseases in individuals from nearly all major angiosperm orders [91]. Furthermore, a single species can infect numerous hosts; for example, *Pu. graminis* has a broad host range, including 365 plant species across 54 genera [92,93]. *Puccinia* spp., which have co-evolved with specific hosts, are further classified as ‘formae speciales’ [91,92]. Among them, three wheat-infecting *Puccinia* species are collectively ranked third [15]: *Pu. graminis* forma speciales (f. sp.) *tritici*, *Pu. striiformis* f. sp. *tritici*, and *Pu. triticina*, causing stem (black) rust, stripe (yellow) rust, and leaf (brown) rust, respectively. These three species are highly significant due to their devastating effects on wheat, a staple food for more than half of the world’s population.

In addition to co-evolving with specific hosts, host jumps (or shifts) have also contributed to the evolution of wheat rust pathogens [91]. Modern polyploid wheat cultivars are believed to have originated from the diploid wild relative *Aegilops speltoides* [94]. The wheat rust pathogen *Pu. triticina* has been frequently reported among commonly cultivated hexaploid wheats and related tetraploid hosts [94]. *Aegilops speltoides* is the only diploid host on which *Pu. triticina* is reported naturally [94], although the disease has been induced on other wild grasses through artificial inoculation [95]. Also, the common ancestral form of *Pu. triticina* is believed to have occurred on the *A. speltoides* [96]. Coalescence analyses suggest that isolates from tetraploid wheat in Ethiopia may represent the oldest derived form of *Pu. triticina* on wheat before the pathogen’s adaptation to the widely grown hexaploid wheats [94,96]. Simultaneously, studies reveal that durum wheat (*Triticum turgidum*; tetraploid) infects *Pu. triticina* isolates in Europe, South America, Mexico, and the Middle East evolved from isolates on hexaploid common wheat [94].

These fungi are dikaryotic, carrying two distinct haploid nuclei within each cell along with the two chromosome sets [97]. Also, these three *Puccinia* species, *Pu. graminis* f. sp. *tritici*, *Pu. striiformis* f. sp. *tritici*, and *Pu. triticina,* were initially believed to reproduce asexually but were later found to complete their sexual cycle using barberry (*Berberis* spp.) as an alternate host [90,91,98,99]. During asexual reproduction, they produce dikaryotic urediniospores on wheat (the primary host) and teliospores when they find suitable alternate hosts, such as *Berberis* spp., during sexual reproduction [90,91,100]. Urediniospores re-infect the host and can also spread long distances through the wind, resulting in wheat rust epidemics, even on a continental scale [101]. The occurrence of sexual and asexual reproduction of these species exhibits a geographic gradient [102]. For instance, in *Pu. striiformis*, natural sexual reproduction occurs frequently in China, Pakistan, and Nepal, leading to recombinant populations, whereas in Australia, Europe, and the United States, only asexual reproduction has been observed, resulting in a clonal population structure [90,102,103]. Therefore, it is believed that these pathogens originated in Asia, particularly near the Himalayan region, and later spread to other parts of the world due to the advent of agriculture [90,103]. Owing to the absence of sexual reproduction within the primary host, new pathotypes have emerged due to frequent introductions or the evolution of new pathotypes through processes such as mutation, somatic hybridization, and parasexuality [94,100,104,105].

In these three clonally reproducing *Puccinia* spp., the development of new races with host resistance is believed to occur through the accumulation of mutations [94,100,103,104]. Fellers et al. [104] observed these mutations occur in both the noncoding and coding regions in the genome of *Pu. triticina*, suggesting that changes are happening in both functional and regulatory areas of the DNA. As a result of these genetic alterations, distinct genetically differentiated groups of *Pu. triticina* would emerge and be observed to have a consistent association with the pathogenicity. Mutations hinder the recognition of pathogen effectors by host resistance (R) genes. In the absence of recognition, these effectors suppress the immune responses of the host and facilitate the infection [103,106]. Successful mutants can emerge as new pathogen races, making previously resistant wheat varieties susceptible. Feodorova-Fedotova and Bankina [107] described how the novel pathogenic races of *Pu. striiformis* have dominated by replacing existing races, acquiring characteristics such as a shorter latent period, extended spore germination, and tolerance to high temperatures compared to the previous races. Mutations occur through single nucleotide polymorphisms (SNPs), small insertions and deletions, larger structural variations, and the movement of transposable elements, as well as taking place in sufficient numbers due to the large size of the rust population [97,101,103,108,109].

While mutations are thought to mainly affect the variations in virulence of wheat infecting *Puccinia* spp., somatic hybridization (parasexuality) also contributes to the emergence of similar virulence traits. Li et al. [110] revealed that the Ug99 strain of *Pu. graminis* f. sp. *tritici*, which emerged in Africa with extreme virulence in the late 1990s, did not arise from mutations in existing strains. The authors found that Ug99 resulted from somatic hybridization, where two dikaryotic *Pu. graminis* f. sp. *tritici* fungal strains fused, exchanging intact nuclei. Furthermore, one of the nuclei of Ug99 strain was found to be identical to an older strain called *Pu. graminis* f. sp. *tritici* 21-0. Zhao et al. [111] stated that there is a high variability in the virulence of *Pu. striiformis* f. sp. *tritici* is attributed to mutation and somatic hybridization, as indicated by previous studies on hyphal fusion, heterokaryosis, germ tube fusion, and genetic recombination between *Pu. striiformis* f. sp. *tritici* strains. Schwessinger et al. [103] also discussed somatic hybridization in *Pu. striiformis* f. sp. *tritici*, noting that it enables the fungus to bypass the initial adaptation stage on wheat, as each haploid nucleus is likely already adapted to wheat. Wang and McCallum [112] reported the occurrence of parasexual recombination in *Pu. triticina* through germ tube anastomosis.

## 5. Evolutionary Journey of *Fusarium graminearum*

*Fusarium graminearum* is a devastating fungal pathogen in the family Nectriaceae (Hypocreales) [113]. This fungus is well known for causing Fusarium head blight disease (improperly called scab), ear rot, and root and crown rot of cereals and other small grains [113,114,115,116], reducing both the yield and quality of crops. Moreover, *F. graminearum* contaminates infested grains with mycotoxins, trichothecenes and zearalenone, which are harmful to human and animal consumption [113,115,116,117]. Pathogenic fungal species closely related to *F. graminearum* and have similar pathogenic behaviors are grouped in the F. graminearum species complex (FGSC). Currently, FGSC comprises about 17 phylogenetically distinct species [118]. *Fusarium graminearum* is believed to have originated earlier in the Asian region than in other parts of the world because Fusarium head blight-resistant plant genotypes are frequently reported in the Asian region, possibly as a result of the long-term coexistence of host and pathogen [119].

*Fusarium graminearum* can infect about 26 plant families, including cultivated and wild plants, particularly in the Poaceae family [120]. Among the cultivated food crops, this pathogen causes diseases in wheat, barley, corn, oats and rice [113]. Wild plants such as weeds and other non-cultivated grasses, as well as forages, also serve as hosts for *F. graminearum* [116,120,121]. Lofgren et al. [116], who worked on *F. graminearum* in American wild grasses, reported that the pathogen might have a long evolutionary history and distinct biochemical interactions with non-cultivated grasses than cultivated crops, suggesting that wild grasses are perhaps the ancestral hosts of this pathogen. Moreover, the pathogens that inhabit most wild grasses are endophytes that show no symptoms [116]. After several years, at the time of the introduction of domesticated grasses by humans, *F. graminearum* had shifted from wild grasses to cultivated grasses, showing symptoms more likely to be a pathogen–host maladaptation than host–pathogen co-evolution [116]. Fulcher et al. [121] showed how well non-cultivated grass species support *F. graminearum* surviving in the field boundaries and during fallow periods. Similar to the shifting from wild species to cultivated crops, *F. graminearum* can also pass through the cultivated hosts. *Fusarium graminearum* isolates from wheat can infect rye, barley, oats, sorghum, triticale, maize, and canola [122]. However, the aggressiveness of the pathogen has been significantly changed after the infection. Therefore, it is evident that the infection of different hosts leads to the emergence of novel pathogenic strains.

Genetic recombination contributes to increasing the genetic diversity in the population and can lead to new variants or lineages that are well-adapted to the host and environment [123]. Therefore, recombination permits efficient co-evolution of host and pathogen by selecting favorable alleles [123,124]. In *F. graminearum* the frequency of sexual recombination is very high [125]. It is suggested that genetic diversity be created in the active regions of its genome, where high recombination rates were observed [123]. In such a way, *F. graminearum* acquired fungicide resistance [125,126,127,128,129] and emerged in more aggressive populations with higher pathogenicity in agricultural fields [130,131,132,133]. However, evidence suggests that HGT may have played a role in the evolution of fungal secondary metabolite biosynthesis in *F. graminearum*, and specific secondary metabolic gene clusters have been horizontally transferred from *Botrytis* or *Cochliobolus* (Syn. *Bipolaris*) species, as these clusters are present in representatives of these lineages Sieber et al. [134].

## 6. Evolutionary Journey of *Fusarium oxysporum*

*Fusarium oxysporum*, a species within the F. oxysporum species complex, belongs to the family Nectriaceae (Hypocreales). *Fusarium oxysporum* is an asexual, cosmopolitan, soil-borne fungus encompassing both pathogenic and non-pathogenic strains [9,135,136]. The plant pathogenic forms of this fungus are known to cause vascular wilt and root rot diseases in more than 120 cultivated crops worldwide [137,138], while non-pathogenic forms are saprophytes [139] and endophytes [140]. Based on their host range, pathogenic strains of *F. oxysporum* are classified into formae speciales [9,136,141,142]. There are over 100 formae speciales [143], each encompassing *F. oxysporum* isolates with similar or identical host ranges [139,144]. Examples include *F. oxysporum* f. sp. *cubense* (infecting bananas), *F. oxysporum* f. sp. *phaseoli* (infecting leguminous plants), *F. oxysporum* f. sp. *ciceris* (infecting chickpeas), *F. oxysporum* f. sp. *lycopersici* and *F. oxysporum* f. sp. *radicis*-*lycopersici* (infecting tomatoes), *F. oxysporum* f. sp. *fragariae* (infecting strawberries), and *F. oxysporum* f. sp. *vasinfectum* (infecting cotton) [135,141,142,145,146,147]. These formae speciales are further categorized into pathogenic races based on their ability to infect specific cultivars of host plants [136,143,148]. For instance, *F. oxysporum* f. sp. *ciceris* has eight known pathogenic races: 0, 1A, 1B/C, 2, 3, 4, 5, and 6, each capable of infecting different chickpea cultivars with varying resistance genes [141,149].

Novel pathogenic races of *F. oxysporum* possibly occurred in agricultural systems either by introducing the pathotypes from elsewhere or local origin [136]. The movement of *F. oxysporum* f. sp. *apii*, the cause of Fusarium yellows of celery, throughout North America most likely originated from a single strain [136]. Also, *F. oxysporum* f. sp. *cubense*, the pathogen responsible for banana wilt, is known to move regionally and between plantations on infected banana cuttings that are used to start new plantations [150]. Moreover, the virulence of pathogenic isolates of native *F. oxysporum* f. sp. *vasinfectum* was found to increase after serial passage on susceptible cotton plants, and this process also quantifies the genetic diversity of the resulting offspring population [147].

In the second scenario, the local origin of new pathogenic *F. oxysporum* races could occur either by evolving from an existing pathogen, which is the classic situation where selective pressure of resistant cultivars encourages the development of more virulent races [135,136], or by emerging from a larger pool of generalist non-pathogenic (and possibly endophytic) *F. oxysporum* strains [136]. If pathogens indeed evolved from non-pathogenic fungi, it is possible that they acquired the pathogenicity and adapted to new environments through genetic variations. In the past, it was believed that *F. oxysporum* reproduces asexually, without (or at least) involvement of sexual reproduction [9,149,150,151]. Although this results in clonal populations, studies have shown the emergence of novel *F. oxysporum* pathogenic races that can overcome host plant resistance through asexual mechanisms such as mutations, HGT, horizontal chromosome transfer (HCT), and parasexual recombination.

In *F. oxysporum*, the accumulation of mutations (such as transposon activity, DNA copy error, etc.) has led to the development of novel genetic variations [9,142,149,150]. del Mar Jiménez-Gasco et al. [149] demonstrated a simple pattern of race evolution in *F. oxysporum* f. sp. *ciceris*, following a stepwise process with few parallel gains or losses. This involves sequential accumulation of mutations to occur virulence in clonal lineages, resulting in races capable of overcoming multiple host plant resistance genes or multiple resistant cultivars. In *F. oxysporum*, HGT has contributed to acquiring virulence factors and other traits, enabling the fungus to adapt to new environments and hosts. For instance, Proctor et al. [152] reported that the fumonisin biosynthetic genes in *F. oxysporum*, which are important for the virulence of the fungus, exhibited low levels of divergence, suggesting that these genes may have been horizontally transferred from a related species or a closely related organism to *F. oxysporum*. Moreover, there is evidence that horizontal transfer of pathogenicity genes, specifically Fusaric acid biosynthetic (FUB) genes and Secreted In Xylem (SIX) genes, in *F. oxysporum* f. sp. *cubense* [145,153].

*Fusarium oxysporum* possesses two types of chromosomes: core and accessory or lineage-specific [154]. All *F. oxysporum* strains have 11 core chromosomes, which carry out necessary housekeeping tasks. Accessory chromosomes, whose numbers vary, aid the fungus in adapting to new environments [155,156,157]. These accessory chromosomes are involved in the HCT [155], which occurs when the haploid nuclei of two germinating spores from different *F. oxysporum* strains fuse, gradually losing core chromosomes from one strain [158]. Host specificity and pathogenicity-related genes in pathogenic *F. oxysporum* are thought to be acquired through these accessory chromosomes via HCT [142]. Ma et al. [155] demonstrated that transferring two accessory chromosomes between different *F. oxysporum* strains led to converting a non-pathogenic strain into a pathogen. Parasexual recombination also contributes to the emergence of new pathogenic strains within the F. oxysporum complex [150,151]. Parasexual recombination occurs between vegetatively incompatible strains of *F. oxysporum*, where genetically distinct nuclei fuse within a single cell, forming stable hybrid cells with new genetic traits [159]. Several studies have demonstrated the occurrence of parasexual recombination in different *F. oxysporum* formae speciales [160,161,162].

However, challenging the previous assumptions that *F. oxysporum* solely reproduces asexually, Fayyaz et al. [163] recently discovered an active sexual cycle in *F. oxysporum* f.sp. *ciceris*.

## 7. Evolutionary Journey of *Blumeria graminis*

*Blumeria graminis* (Syn. *Erysiphe graminis*) causes powdery mildew disease in various grasses and cereals [164,165]. This obligate biotroph belongs to the phylum Ascomycota, class Leotiomycetes, order Erysiphales, and family Erisyphaceae [166,167]. *Blumeria graminis* causes devastating damage to wheat and barley, and this fungus also represents a model organism for studying biotrophic pathogen systems [15]. Although the order Erysiphales consisted of numerous species causing powdery mildew in various angiosperms, *Bl. graminis* is the sole causal agent for powdery mildew of cultivated and wild poaceae hosts (particularly sub-family Pooideae) [165,168,169]. This single species has been strictly specialized into particular host plants, limiting their natural infection to a single host genus [168]. Based on this host specialization, *Bl. graminis* has been classified into ‘forma speciales (f. sp.)’ such as *Bl. graminis* f. sp. *tritici*, f. sp. *hordei*, f. sp. *secalis*, and f. sp. *avenae* that are specialized in wheat, barley, rye and oat, respectively; and four f. sp. specialized in wild grasses such as *Dactylis* spp. *Agropyron* spp. and *Poa* spp. or *Bromus* spp. [164,170]. Menardo et al. [171] later introduced two formae speciales, *Bl. graminis* f. sp. *triticale* and f. sp. *dicocci* are specialized to triticale and wild emmer (tetraploid) wheat, respectively.

Unlike other formae speciales described previously, studies have shown that most of these formae speciales have not co-evolved with their specific hosts, as the divergence of hosts and pathogens is not simultaneous [170,172,173]. In this context, the pathogen’s divergence appears to be more recent than that of its host [174]. Therefore, it is believed that the same pathogen has later adapted to a new host through the host shift or jump rather than diverging from a common ancestor simultaneously with the host [172]. Similarly, these formae speciales had not undergone co-speciation, as multiple host-jumpings to phylogenetically distinct hosts occurred during the evolution of the fungus [168]. However, some lineages, such as *Bl. graminis* f. sp. *poae* and hordei, have co-evolved with their hosts, while others have shown host jumps [165]. For *Bl. graminis*, it is recommended to restrict the concept of formae speciales to the definition of isolates growing on the same host without directly attributing evolutionary implications due to the high gene tree inconsistency in the recently introduced formae speciales [165].

*Blumeria graminis* exhibits facultative reproduction [175], where sexual reproduction occurs only at certain conditions, particularly when the conditions are not favorable. For instance, *Bl. graminis* is found to undergo sexual reproduction against the host senescence [169] and extreme climatic conditions [176]. During sexual reproduction, hyphae of opposite mating types fuse together develop into chasmothecium (fruiting body) that contains ascospores [169]. These chasmothecia also function as overwintering structures, allowing them to survive during the winter and act as sources of inoculum for subsequent warmer seasons [176]. However, *Bl. graminis* is reported to reproduce sexually during dry summers in Morocco [177]. In *Bl. graminis*, sexual reproduction has led to genetic variations through mutations, migration and recombination, forming novel aggressive pathotypes [176].

Studies have shown that sexual reproduction can occur even among formae speciales, leading to the development of new pathogenic progenies [165,168]. For instance, formae speciales *tritici* and *agropyri* can produce fertile progeny [178]. *Blumeria graminis* f. sp. *triticale* originated from the hybridization of *Bl. graminis* f. sp. *secalis* and *Bl. graminis* f. sp. *tritici* [171]. However, the emergence of *Bl. graminis* f. sp. *triticale* is more likely due to the adaptation of *Bl. graminis* f. sp. *tritici*, which has a broad host spectrum, to triticale, rather than genetic hybridization of geographically isolated populations of *Bl. graminis* f. sp. *secalis* and *Bl. graminis* f. sp. *tritici* [164]. Wicker et al. [179] suggested that the gene pool of *Bl. graminis* f. sp. *tritici* contains ample genetic diversity necessary for adapting to various host species. Their results on characterization of genetic diversity suggested that this swift adaptation is due to the presence of diverse groups of genes originating from haplogroups that existed before wheat domestication. Furthermore, the transition from wild tetraploid to domesticated hexaploid wheat appeared not to have diminished genetic diversity in *Bl. graminis* f. sp. *tritici*, implying that this diverse haplotype pool provides substantial genetic potential for pathogen variation, facilitating swift adaptation.

The emergence of the novel *Bl. graminis* pathotypes are found to be influenced by the genetic structure of the host population, particularly concerning host resistance genes (R genes). Jensen et al. [180] studied the spatial distribution and evolution of *Bl. graminis* f. sp. *hordei* populations in Morocco, and discovered that selection by the host population led to the evolution of new and distinct pathotypes. The cultivation of genetically diverse traditional barley varieties in smallholder farms resulted in weaker selection pressure on *Bl. graminis* f. sp. *hordei*, leading to higher diversity in virulence genes and combinations. Conversely, in Europe, where barley cultivation relies on genetically uniform cultivars with consistent R-gene combinations, strong selection pressure on *Bl. graminis* f. sp. *hordei* promoted the rapid evolution of new pathotypes. Marshall et al. [181] modelled the effects of cultivar mixtures on the evolution of the aggressiveness of *Bl. graminis* f. sp. *hordei*. The developed model indicated that the growing of cultivar mixtures slows the evolution of the pathogen as well as decreases disease outbreaks by preventing the emergence of new pathotypes.

In addition to the monoculture of susceptible cultivars, long-term use of fungicides having a single mode of action has also been reported to cause the emergence of novel powdery mildew pathotypes. Tucker et al. [182] showed that the widespread application of DeMethylation Inhibitor (DMI) fungicides resulted in the selection of *Bl. graminis* f. sp. *hordei* isolates carrying specific mutations (Y137F and S524T) that are associated with resistance factors of certain fungicides. Godet and Limpert [183] reported that *Bl. graminis* f. sp. *tritici* in France exhibited increased resistance to DMIs and morpholines between 1993 and 1996, attributing this multiple resistance to the recombination of respective resistance genes. Furthermore, they found that the decrease in morpholine usage after 1992 aligns with an increase in morpholine resistance. In Morocco, reduced fungicide usage and increased recombination are expected to sustain a high diversity of *Bl. graminis* f. sp. *hordei* pathotypes, offering the potential for rapid evolutionary changes in the pathogen population [180].

## 8. Evolutionary Journey of *Zymoseptoria tritici*

*Zymoseptoria tritici* (Syn. *Mycosphaerella graminicola*) causes septoria tritici blotch, a significant disease of wheat with high economic importance and a widespread global impact on wheat production. This necrotrophic ascomycete belongs to the order Dothideales [184,185]. Primarily, *Z. tritici* specialized in infecting cultivated bread and durum wheat (*Triticum aestivum* L. and *T. turgidum*) [184,185]. However, studies have shown that *Z. tritici* can infect other grass species, such as *T. durum*, *T. dicoccum*, and *T. compactum*, providing primary inoculum sources for the pathogen [186]. *Zymoseptoria tritici* is believed to have originated in the Fertile Crescent from sympatric ancestral populations infecting wild grasses during the domestication of wheat [187]. Comparative genomics analysis of *Z. tritici* and its closest known progenitor species (*M. graminicola* S1) suggests that the significant differentiation of genomes might have occurred either gradually over an extended period or rapidly as a consequence of host domestication. Furthermore, the rapid divergence among pathogen lineages is believed to result from the co-evolution of *Z. tritici* with wheat, leading to the pathogen becoming specialized for its host [188,189]. Studies have shown several mechanisms underlying this new host adaptation and speciation of *Z. tritici*. These include genome plasticity [190], structural rearrangements in the small dispensable chromosomes, and strong positive selection on protein-coding genes [188,191].

The high genome plasticity, reflecting the ability of the *Z. tritici* population to undergo genomic changes, has enabled rapid adaptation and overcoming of adverse biotic and abiotic conditions in the wheat environment [185]. Goodwin et al. [190] sequenced the genome of *Z. tritici*, uncovering 21 chromosomes. Eight of these chromosomes are found to be dispensable, having the ability to be lost without causing visible effects on the fungus, potentially varying in number among field and progeny isolates. These dispensable chromosomes are suggested to have originated through ancient horizontal transfer from an unknown donor, followed by extensive recombination. The remaining chromosomes formed the core, consistently present in both field and progeny isolates, likely harboring essential genes crucial for survival, thus rendering them indispensable. In addition to variations in dispensable chromosome numbers, *Z. tritici* exhibited genomic plasticity with translocation of chromosome sections, chromosome length polymorphisms, and chromosome copy number polymorphisms [192].

Random drift, natural selection, mutation, gene flow, and sexual recombination are the major evolutionary forces influencing the population dynamics of *Z. tritici*. The empirical data revealed that random drift and natural selection decrease genetic variation, whereas mutation, gene flow, and sexual recombination contribute to genetic variation in the fungus [193]. The high genetic variation in *Z. tritici* has led to rapid evolution in response to selective pressures, particularly resistant wheat cultivars and the continuous use of fungicides. For instance, the resistance of some wheat cultivars to septoria tritici blotch disease has significantly deteriorated due to the natural selection of *Z. tritici* strains with specific virulence, suggesting the widespread use of major resistance genes is likely to result rapid emergence of new virulent strains [194,195]. Therefore, to achieve long-lasting disease control, it may be necessary to consider using moderately resistant cultivars, cultivar mixtures, and/or implementing resistance gene rotations [195,196,197]. The effectiveness of certain fungicide groups has also been reported to decline due to mutations in pathogen target genes [198,199,200], intra-genic recombination [201], and the rapid evolution of specific target proteins [202] in response to the widespread use of these fungicides.

*Zymoseptoria tritici* undergoes both sexual and asexual reproduction, giving rise to ascospores and pycnidiospores, respectively [203]. The sexual ascospores are airborne and hypothesized that they act as the primary propagules for long-distance dispersal, covering distances of up to hundreds of kilometers. In contrast, the asexual conidia are believed to be dispersed over much shorter spatial scales, typically within a few meters, through rain-splash [204]. Furthermore, ascospores play a significant role in over summering to survive during the non-host seasons, while pycnidiospores are primarily responsible for disease development during the growing season [5,203]. Engaging in both sexual and asexual reproduction has enabled *Z. tritici* to thrive in dynamic environments. The active sexual cycle creates high genetic diversity within the population [5,193,205], while asexual reproduction, combined with natural selection and gene flow, ensures the maintenance and rapid dissemination of allele combinations [193]. A field study evaluating the roles of sexual and asexual reproduction in *Z. tritici* during an epidemic cycle found that sexual reproduction among introduced isolates resulted in new recombinants [206]. These recombinants have further displayed increased virulence and fungicide tolerance, demonstrating the role of sexual reproduction in enhancing the pathogen adaptability.

## 9. Evolutionary Journey of *Colletotrichum* spp.

The genus *Colletotrichum* includes a number of devastating plant pathogens that cause diseases in a wide variety of woody and herbaceous hosts [207,208,209]. Almost every plant family cultivated is believed to be susceptible to one or more species of *Colletotrichum* [208]. *Colletotrichum* spp. have been reported to occur in over 3400 host species [210], including a wide range of cultivated and wild plants [211,212,213,214]. Fruit plants such as strawberry, mango, citrus, avocado and banana are particularly affected by this genus. In contrast, cereals, including corn, sugarcane, sorghum, and other cultivated crops such as coffee, onion, eggplant, etc., are also affected [207,208].

*Colletotrichum* species utilize a wide array of strategies for colonizing hosts and acquiring nutrients, including necrotrophism, hemibiotrophism, latency or quiescence, endophytism, and saprotrophism [215,216]. Virtually all major groups of angiosperms are known to host endophytic colonies of Colletotrichum [207]. In response to environmental signals such as host senescence, injury, or alterations in plant physiology [217,218,219,220], these endophytic *Colletotrichum* species can adapt to either a saprotrophic [221,222] or pathogenic lifestyle [217,218,219,220,223,224]. This implies that the endophytic phase is a shared trait among all species, and alternative lifestyles may have evolved from it [225]. However, there is evidence of pathogenic strains transforming into beneficial endophytes that confer mutualistic advantages, including host protection against highly virulent strains of the same species and other plant pathogens like *Fusarium* [226,227]. Furthermore, the single disruption events in pathogenicity genes can shift a pathogen from causing disease to becoming a beneficial endophyte with protective functions [228,229,230].

Numerous theories have been proposed to explain the emergence of new *Colletotrichum* pathogens in agroecosystems. In Brazil, *Colletotrichum* species appear to have co-evolved with cashew (*Anacardium* spp.). This was evident from the greater diversity of *Colletotrichum* species associated with cashew anthracnose, likely due to Brazil being a primary center of diversity for *Anacardium* spp. [214]. The authors further described that the domestication of wild cashew plants reduced the effectiveness of certain compounds that were originally employed as chemical defenses against plant pathogens. As a result, the domesticated cashew plants have become more susceptible to various *Colletotrichum* species. The presence of a large number of pathotypes exhibiting a high degree of aggressiveness diversity in *C. lindemuthianum* is believed to be a result of the co-evolutionary relationship between the pathogen and its host, Common bean [231]. Similarly, Crouch et al. [213] also described that the divergence of species within the graminicola species complex appears to have co-evolved with the Poaceae family, which includes grasses. However, Baroncelli et al. [208] suggested that there is little to no indication of a co-evolutionary relationship between host plants and *Colletotrichum* pathogens, as evidenced by the vulnerability of cultivated fruits like strawberries and olives to various members of the C. acutatum species complex.

The emergence of novel *Colletotrichum* pathogens has also been proposed to occur through host shifts. Silva et al. [232] reported that the emergence of *C. kahawae* in Arabica coffee is most likely due to a recent host jump and subsequent host specialization. Authors describe that the ancestral population of *C. kahawae* most likely emerged from hosts other than *Coffea* spp. and then jumped to the newly arrived *Coffea arabica* plants in Angola. Crouch et al. [233] proposed that habitat transformation (alteration of the ecosystem, i.e., due to human activities) is another plausible factor contributing to population divergence, particularly in turfgrass anthracnose pathogen *C. cereale*. The authors discovered that *C. cereale* is specialized in the unique turfgrass environment typically found on golf courses, hindering its movement from cereal crops and prairie grasses to turfgrass areas. Since the fungus has adapted to the specific conditions of the turfgrass environment, this specialization may result in population divergence in *C. cereale*. Moreover, Doyle et al. [212] proposed that the genetic diversity of *C. gloeosporioides* is influenced by both habitat and host. Their study revealed that the isolates from agricultural landscapes were more closely related to each other than to isolates from wild landscapes, suggesting that the pathogen has undergone a recent shift from wild to agricultural hosts.

*Colletotrichum* species are generally not known for frequent sexual reproduction, and for most species, the sexual morph has not yet been described in nature [234]. This limited sexual reproduction has important implications for the genetic diversity and evolution of *Colletotrichum*. However, evidence has been presented by Crouch et al. [235] for sexual recombination in the fungus *C. cereale*, shedding light on the role of repeat-induced point (RIP) mutation in this process. The genome of *C. tanaceti* contains numerous repetitive elements that may result in the rapid generation of new genetic variations via RIP mutations [236]. Therefore, the pathogenicity genes present within these RIP-affected regions have a higher tendency to evolve novel pathogenicity genes, making the fungus more virulent.

Several asexual mechanisms of genetic variation have been suggested for *Colletotrichum* species. For instance, Castro-Prado et al. [237] demonstrated the occurrence of parasexual recombination among the vegetative compatible mutants of *C. lindemuthianum* under laboratory conditions. However, there is a lack of evidence for this phenomenon in nature [234]. Genetic variations among *Colletotrichum* spp. have also been found to occur through HGT, playing a major role in pathogenicity and niche adaptation [236,238]. Scientists have discovered genes from bacteria [216,238] and plants [239] have also been horizontally transferred to the *Colletotrichum* ancestors, in addition to fungus-to-fungus horizontal transfer. In particular, genes related to amino acid, lipid, and sugar metabolism, as well as lytic enzymes, have been identified as horizontally transferred genes in *Colletotrichum* [238]. The horizontal transfer of accessory chromosomes has also been demonstrated to contribute to developing novel pathogenicity and virulence in *Colletotrichum* species [240,241]. Additionally, Roca et al. [242] demonstrated the conidial anastomosis, a process involving the fusion of conidia and the exchange of genetic materials between two fungal species, as a novel mechanism for genetic variation in the genus *Colletotrichum*. The authors reported that conidial anastomosis tubes readily form between conidia within acervuli during conidiogenesis in *C. lindemuthianum* and *C. gossypii*. These tubes facilitate the movement of cytoplasm and organelles, resulting in conidia that may lack nuclei or contain more than one nucleus.

The gains and losses of certain genes or gene families have also been found to have contributed to *Colletotrichum* evolution [209,213,216,236]. Genome-wide gene family evolutionary analyses revealed that a range of gene families significantly expanded, including genes related to various biological functions such as biosynthesis, oxidoreduction, detoxification, transport, and *Colletotrichum* genus-specific genes related to infection ability and adaptation of the fungus [209,216]. On the other hand, some genes were lost, such as genes related to RTA1 proteins, which may play a role in detoxification in the monocot-specializing graminicola complex [216]. In the context of *Colletotrichum* species adapting into specific hosts, effector genes have also been found to play a crucial role. Comparative genomics studies have shown that every *Colletotrichum* species possesses a significant number of unique effector gene candidates, emphasizing their importance in host adaptation [243].

## 10. Insights to Mitigate Pathogen Evolutionary Dynamics in Agro-Ecosystems

The study of the evolutionary journey of these economically important fungi revealed that they have been successfully established in agricultural ecosystems, regardless of host ranges. Some species, such as *Bo. cinerea*, *Puccinia* spp., *F. oxysporum*, and *Colletotrichum* spp., infect a wide variety of hosts, while others are restricted to a few hosts, particularly within a single family, such as *Py. oryzae*, *F. graminearum*, and *Bl. graminis*, or a single genus, such as *Z. tritici*. This indicates that host range does not necessarily affect the ability of pathogens to adapt and evolve successfully. For some pathogens, like *Bo. cinerea*, the host range is still uncertain due to insufficient discovery of wild hosts. However, it is immensely important to identify wild hosts because most pathogenic fungi originated in wild forms and then transferred into domesticated hosts. Additionally, the cultivated lands evade the wild habitats and more virulent forms could be easily shifted to cultivated plants resulting in sudden outbreaks [4].

**Figure 2:**
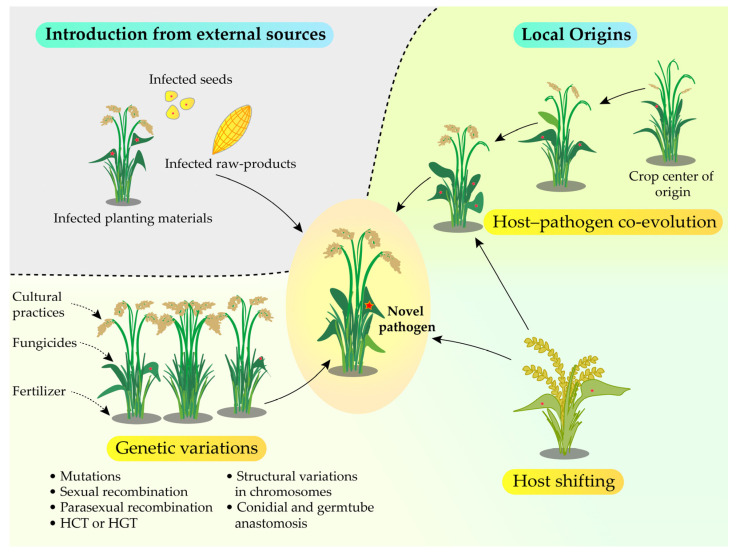
An illustration depicting the emergence of novel pathogenic fungi in agricultural ecosystems. Phytopathogenic fungi can evolve either through the introduction from external sources or through local origins, which involve processes such as host-pathogen co-evolution, host shifting, and genetic variations within existing pathogenic strains.

Perhaps other selected pathogens also have evolved and established in agricultural ecosystems either through introduction from elsewhere or from local origins, similar to what has been described for *F. oxysporum* [136] (Figure 2). Based on the evidence from the studied pathogens, such introductions can occur on both regional and continental scales through planting materials and agricultural produce. These risks could be minimized by implementing quarantine regulations and phytosanitary measures [11,244]. Locally, pre-existing pathogens can emerge as new variants through co-evolution with host plants, shifting to other hosts, or genetic variations. Among these, it was evident that genetic variations are the primary force driving the evolution and adaptation of emerging pathogens. Similarly, Woolhouse et al. [245] also described that the likelihood of a pathogen evolving or adapting to new hosts or environmental conditions largely depends on its capacity to acquire the necessary genetic variations. Although sexual reproduction generates frequent genetic variations through recombination, most pathogens discussed in this review do not often reproduce sexually. Instead, these pathogens primarily generate genetic variations through asexual mechanisms, including mutations and parasexual recombination (somatic hybridization). Additionally, other mechanisms such as HGT, HCT, structural variations in chromosomes, conidial, and germ tube anastomosis were also found to contribute to the creation of genetic variation among pathogen populations. Therefore, knowing these mechanisms and discovering possible ways to interrupt them is crucial to preventing sudden outbreaks caused by novel pathogen variants.

The emergence of new pathotypes in agricultural landscapes was also found to be influenced by certain farming practices. These include the mono-cropping of highly resistant cultivars on a large scale and the continuous long-term use of fungicides with the same mode of action. This could result in the rendering of plant resistance, either through the loss of avirulence genes or mutations in pathogenic fungi. Furthermore, fungicide resistance in pathogen populations often occurs through target site modifications due to mutations and genetic recombination via sexual reproduction in pathogens. Therefore, using moderately resistant cultivars, cultivar mixtures, resistance gene rotations, and application of fungicides with different modes of action is recommended. This will minimize the selective pressure imposed by humans on the evolution of novel pathotypes. At the same time, it is advisable to minimize the presence of weeds belonging to the same family as the crop, as they can serve as alternative hosts for pathogens and potentially lead to the emergence of new pathogen strains through host shifting. Stubble can also serve as a resting place for pathogens and provide an inoculum source for subsequent seasons.

## 11. Techniques Used to Understand Evolutionary Trends in Plant Pathogenic Fungi

The study of pathogen evolution in laboratory and natural environments has become more feasible with genomics, especially due to the reduced costs of next-generation sequencing technologies [246,247]. These techniques are employed to precisely measure mutation rates, identify genetic targets and the dynamics of natural selection, explore the relationship between genetic and phenotypic changes, and supply data to test long-standing evolutionary theories [165,246]. There are two approaches to whole-genome analysis of fungal pathogens: de novo sequencing for species that have not been sequenced before and re-sequencing for species with an existing high-quality reference assembly [247]. The first approach provides new genome assemblies, facilitating the study of emerging pathogens causing recent outbreaks [247,248]. The latter allows tracing the evolution of pathogens over time, tracking the emergence of novel genotypes and the spread of virulent strains [66,247,249].

In recent years, there has been an increase in the number of publicly available genome sequences [209,250]. For instance, a total of 18,414 fungal genomes, including 5886 reference genomes and 5102 annotated genomes, are publicly available in the National Center for Biotechnology Information (NCBI; https://www.ncbi.nlm.nih.gov/datasets/genome/; accessed on 31 July 2024). This has enabled the comparison of sequences and the exploration of similarities and differences in pathogen-host interactions, thereby uncovering the genetic mechanisms involved in pathogen evolution [171,216,247,251,252,253,254]. This approach is beneficial for uncovering ancient evolutionary events and understanding the broad evolutionary trajectories of pathogens [179,241,250].

Gene content alone cannot fully explain differences in pathogenicity between fungal species, as identical gene contents can still result in varying levels of pathogenicity [252]. Therefore, functional genomic approaches (e.g., transcriptomics) are essential for identifying gene expression and function differences. Transcriptomics enables the analysis of gene expression profiles under different conditions, such as during pathogen infection or environmental stresses, revealing how pathogens adapt and evolve [255,256,257,258]. Furthermore, by identifying virulence factors and adaptive strategies, transcriptomics provides insights into host-pathogen interactions, including host specificity and co-evolution [258,259]. Comparative transcriptomics across strains or species helps trace evolutionary trajectories [260,261], while studies of stress responses and regulatory changes highlight how pathogenic fungi adapt to challenges such as fungicides or plant defenses [262,263,264].

Population genomics is another invaluable tool for studying the evolution of fungal pathogens, offering comprehensive insights into the more recent and ongoing evolutionary processes [250,265]. This approach examines genetic variation within and between populations of a single species to understand how pathogens adapt and evolve in response to contemporary selection pressures, including those imposed by agricultural practices [85,170,250,266]. Genome-wide association studies (GWASs), quantitative trait locus (QTL) mapping, and genome scans are powerful tools used in population genomics to study the evolution of fungal pathogens [250]. The abovementioned three methods help to identify genetic variations associated with specific traits, such as virulence, fungicide resistance, and host adaptation, providing insights into the mechanisms driving pathogen evolution [194,267,268,269,270,271,272].

## 12. Conclusions

Like all living organisms, fungal pathogens continuously evolve, developing traits such as increased virulence, adaptation to new environmental conditions, or resistance to fungicides. Various factors, including genetic variations, natural and anthropogenic selective pressures, and population dynamics, drive this evolutionary process. This review described the evolutionary trends observed in eight economically important fungal pathogen groups, highlighting their potential to generate novel pathogen variants. It may not be feasible to halt pathogen evolution completely; however, proactive management strategies could help slow down its rate. Farmers can create less favorable conditions for the proliferation of virulent pathogen strains by implementing integrated approaches that combine different control measures rather than relying on single methods for extended periods. Advances in molecular biology, genomics, and bioinformatics have revolutionized our understanding and ability to study plant pathogens. These advancements enable research to monitor how fungal pathogens change over time and to develop sustainable strategies in advance to mitigate the impact of new diseases, ultimately contributing to global food security.
